# Patterns and Predictors of Recovery from Poor Health Status Measured with the Chronic Obstructive Pulmonary Disease (COPD) Assessment Test in Patients with Stable COPD: A Longitudinal Study

**DOI:** 10.3390/jcm8070946

**Published:** 2019-06-29

**Authors:** Francesc Medina-Mirapeix, Roberto Bernabeu-Mora, Maria Piedad Sánchez-Martínez, Mariano Gacto-Sánchez, Rodrigo Martín San Agustín, Joaquina Montilla-Herrador

**Affiliations:** 1 Department of Physical Therapy, University of Murcia, 30100 Murcia, Spain; 2 Research group Fisioterapia y Discapacidad, Instituto Murciano de Investigación Biosanitaria-Virgen de la Arrixaca (IMIB-Arrixaca), 30120 El Palmar Murcia, Spain; 3 Department of Pneumology, Hospital General Universitario J M Morales Meseguer, 30008 Murcia, Spain; 4 Department of Physical Therapy, EUSES University School, University of Girona, 17190 Salt Girona, Spain; 5 Department of Physical Therapy, University of Valencia, 46010 Valencia, Spain

**Keywords:** health status, COPD, CAT, transitions, recovery, handgrip strength, non-smoker

## Abstract

Recent recommendations for chronic obstructive pulmonary disease (COPD) suggest that evaluation and management should focus on patient health status. Despite the frequency of poor health status and its negative impact on patients with COPD, little is known about how poor or non-poor health status persists and/or remits over time or what factors might predict recovery from a poor health status. The aim was to determine the likelihood of transitioning between poor and non-poor health status in patients with stable COPD followed for 2 years and to investigate factors that might predict recovery from poor health status. We prospectively included 137 patients with stable COPD (mean age, 66.9 years ± 8.3). Health status was measured at baseline and after 1 and 2 years with the COPD assessment test (CAT). Higher scores indicated worse health status, and 10 was the cut-off score for discriminating between non-poor and poor health status. The likelihoods of annual transitions to new episodes and recovery were calculated. We evaluated demographic, non-respiratory, and respiratory variables as potential predictors with generalized estimating equations. At baseline, 37 patients (27%) reported non-poor health status. Within the group of patients displaying poor health status at the beginning of the year, 176 annual transitions were identified during the study period: 15.9% were transitions to recovery from poor health status. In contrast, of the 70 transitions from a starting non-poor health status, 32.4% worsened. Predictors of transitions to recovery were: current non-smoker status (odds ratio (OR) = 3.88; 95% confidence interval (CI): 0.64–5.54) and handgrip strength (OR = 1.08; 95% CI: 1.00–1.16). This study suggests that self-reported health status, measured with the CAT, has a dynamic nature in patients with COPD. Annual transitions towards recovery from poor health status are most likely among current non-smoking patients and those with high handgrip strength.

## 1. Introduction

Traditionally, chronic obstructive pulmonary disease (COPD) has been characterized as mainly a lung disease, but in recent years, it has been defined as a multisystem disease. COPD has multiple effects beyond lung function, including reductions in health status, exercise capacity, and functional status, among others [1]. The most recent recommendations of the Global Initiative for Chronic Obstructive Pulmonary Disease (GOLD) indicate that disease evaluation and management should focus on the patient’s health status [2], since a poor health state has been identified as a predictor of exacerbations [3] and mortality [4]. Given the importance of health status in patients with COPD, GOLD proposed that COPD should be evaluated with the COPD assessment test (CAT). In this simple, reliable questionnaire, patients with COPD rate health-related items, and higher scores indicate worse health status. An overall score of 10 is the cut-off for discriminating between non-poor and poor health status for COPD patients [2,5,6]. Based on this cut-off score, several studies have determined that the prevalence of poor health status (CAT ≥ 10) varies from 56.8% to 77.8% among patients with COPD [1,7]. Despite the frequency of poor health status and its negative impact on patients with COPD, little is known about how poor or non-poor health status persists and/or remits over time or what factors might predict recovery from a poor health status.

Studying the determinants of health status in COPD is of interest, because determinants can reveal potentially modifiable factors that might minimize the impact of the disease, and thus, improve the patient’s ability to cope with the consequences of this disease [7]. Previous studies have shown a cross-transversal association between poor health status (CAT ≥ 10) and various pulmonary and non-pulmonary factors, such as forced expiratory volume in 1 s (FEV1), history of smoking, dyspnea [8,9] and exercise ability, among others [10]. Nevertheless, predictors of poor health status might differ from factors that predict recovery from poor health status in patients with COPD. 

The present study aimed to determine the likelihood of transitioning between states of poor and non-poor health, measured with the CAT, in patients with stable COPD that were followed for 2 years. We also investigated several factors for their ability to predict recovery from poor health status.

## 2. Materials and Methods

### 2.1. Study Design and Participants 

In this longitudinal study, patients with stable COPD were prospectively recruited from an outpatient pulmonary service at Morales Meseguer Hospital, Murcia, Spain, during the period of 2015–2017. All study participants provided written informed consent, and the study protocol was approved by the Institutional Review Board of the hospital (the Ethics Committee of Clinical Research of the General University Hospital; approval number: EST-35/13, date of approval: 22 July 2013). Patients were included in this study when they fulfilled the following criteria: a diagnosis of COPD, according to the Global Initiative for COPD (GOLD) recommendations (i.e., a post-bronchodilator ratio of FEV1/forced vital capacity post-bronchodilator ratio of <70%) [2], in stable stage (without exacerbations in the previous 6 weeks) and aged between 40 and 80 years. Patients were excluded when they displayed an unstable cardiac condition within 4 months of the start of the study; cognitive deterioration; or inability to walk. Over a 1-year period, a consecutive sample of eligible patients was identified on a rolling-basis, based on patient health examinations. A pulmonary physician assessed eligibility for inclusion among all patients with stable COPD that attended follow-up visits. Patients received examinations at baseline and annually for 2 years. The initial cohort included 137 patients.

### 2.2. Measurements

Study data were obtained in patient interviews conducted at baseline (T0) and at 1 year (T1) and 2 years (T2) after study initiation. Baseline data on sociodemographic, non-pulmonary, and pulmonary variables were obtained for analysis as possible predictors of transitions between states measured with the CAT (poor or non-poor health status). Patient interviews were conducted by a pulmonologist. For follow-up visits, research staff contacted patients 1–2 weeks before each follow-up time to determine if they were in a stable stage within the previous 6 weeks. The interview was conducted within 4 weeks of the due date. Subjects that were not interviewed within this time interval were removed from the study at that time point.

### 2.3. Outcome Measure

We used the CAT to measure health status of patients with COPD [11]. We selected this disease-specific questionnaire vs. other generic questionnaires, such as the SF-36, because usually the latter are less responsive to change than the former. The CAT is a simple, short, sensitive questionnaire for evaluating and quantifying the impact of COPD symptoms on patients’ health. The CAT encompasses eight items measuring different domains of respiratory health, including cough, phlegm, chest tightness, fatigue climbing stairs, household activities, confidence/security in leaving home, sleep quality, and energy. The resulting score ranges from 0 to 40, indicating the best and worst health status, respectively [5]. For this study, we used the score of 10 as the criterion to define poor health status [2,5,6]

### 2.4. Baseline Measures

We selected 15 variables among covariates identified in literature research, which showed potential associations with either health status for COPD patients or CAT scores. These variables were classified into three domains: sociodemographic, non-pulmonary, and pulmonary.

The sociodemographic variables included age (years) and sex. The non-pulmonary variables included body mass index (BMI, kg/m^2^), number of comorbidities, presence of heart disease, symptoms of depression, low physical activity (according to the index of frailty) [12], handgrip strength, a six-minute walk test (6MWT), and a 5-repetitions sit-to-stand (5STS) test. Pulmonary variables included smoking pack-years, smoking status (i.e., smoker vs. current non-smoker), the level of dyspnea, total number of moderate and severe exacerbations in the previous year, and the percent of forced expiratory volume in 1s (FEV1) compared to the predicted value. 

These variables were obtained as follows: patients were measured and weighed to obtain BMI; the patient’s medical history was reviewed to determine smoking pack-years; current non-smoker; the number of comorbidities; the number of exacerbations (moderate defined as use of corticosteroids and/or antibiotics; severe defined as requiring hospitalization); and the presence of heart disease. Physical activity was assessed with a reduced version of the Minnesota Leisure Time Physical Activity Questionnaire [13]. Low physical activity was defined as the lowest quintile of kcals reported, according to the gender of each participant [12]. Handgrip strength was assessed on the dominant side with a handgrip dynamometer (KERN MAP 80K1, KERN & SOHN GmbH Balingen, Germany). The 6MWT was performed indoors, along a straight flat, 30‑m walking course, supervised by 2 well-trained nurses (with a mean of 19 years of experience), according to the American Thoracic Society guidelines [14]. The 5STS test required participants to rise from a chair with their arms across their chest, then sit back down, repeating five times [15]. The 5STS test scores range from 1 to 4; scores < 2 points indicate poor performance [16]. The level of dyspnea associated with activity was measured with the modified British Medical Research Council (mMRC) questionnaire [17]. Depression was assessed with the depression subscale of the Hospital Anxiety and Depression Scale (HADS). A total score equal to or greater than 11 indicated a diagnosis of probable depression [18]. Pulmonary function was assessed with spirometry, performed with a Master Scope Spirometer (version 4.6; Jaeger, Würzburg, Germany), according to the American Thoracic Society guidelines [19].

### 2.5. Statistical Analysis

Baseline characteristics were assessed with descriptive statistics. CAT status was calculated annually. Among participants with non-poor health status (CAT < 10) at baseline, we calculated the percentage that displayed poor health (CAT ≥ 10) in at least one of the two subsequent measurements. Conversely, among participants that displayed poor health status at baseline, we calculated the percentage that displayed health recovery in subsequent measurements.

We examined data on repeated binary measures across all the available 1-year transitions recorded during the 2-year study period. A 1-year transition was defined as an observed difference in health status between a given year and the following year. These data were not available when a participant dropped out or died before the following year. Next, we applied generalized estimating equations to fit a repeated measures logistic regression using the annual transitioning status from poor health to recovery status as the dependent variable to determine the effects of potential predictor variables. For these analyses, several models were constructed. Model 1 included only the sociodemographic variables and BMI; model 2 included each non-pulmonary and pulmonary variable, after adjusting for age, sex, and BMI; model 3 was simultaneously adjusted for all significant (*p* < 0.10) sociodemographic, non-pulmonary, and pulmonary variables. All these models provided odds ratios for recovery of health status measured with the CAT. Unfortunately, we did not run the same analysis for transitions from non-poor to poor health status because these available 1-year transitions were too low. All analyses were performed with the Statistical Package for the Social Sciences (SPSS) version 19.0 (IBM SPSS, Chicago, IL, USA).

## 3. Results

### 3.1. Participants

At baseline, we included 137 patients with a mean age of 66.9 years (87.6% males): all were COPD patients with a history of smoking (smokers or ex-smokers of at least 10 pack-years) and three patients (2.2%) had concurrent asthma. Of these, 100 (73.0%) subjects were in a poor health state with more depression, lower physical activity, shorter 6MWT, and worse 5STS performance that those in non-poor health status. Moreover, patients in a poor health status smoked more pack-years, had more severe dyspnea, experienced more exacerbations, and had a lower percentage FEV1 compared to those in non-poor health status (Table 1).

During the follow-up period, 127 (92.7%) patients remained in the study at T1, and 119 (86.9%) patients remained at T2. Of those lost to follow-up at T2, six (4.3%) patients died, eight (5.8%) patients dropped out due to lung cancer, and four (2.9%) patients chose not to continue. No patients were removed due to an unstable stage or an exacerbation that occurred close to any follow-up visit. 

### 3.2. Rates and Probabilities of a Transition at 1 Year

**Figure 1:**
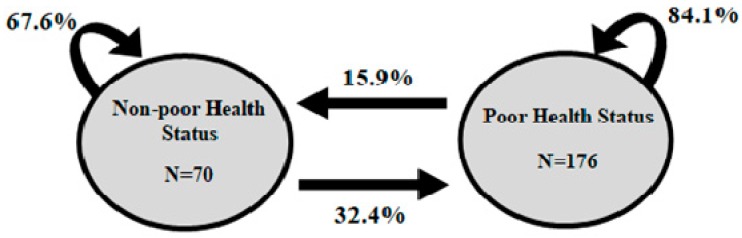
One-year probabilities of a transition between poor and non-poor health status. Circles represent a single state for a 1-year period at the beginning. Straight arrows represent the probability of changing to another state; curved arrows represent continuing in the same state for another 1-year period.

During the 2 years of follow-up, a total of 246 annual transitions occurred between poor and non-poor health status; of these, 176 were transitions to recovery from a poor health status. Based on these transitions, we calculated the 1-year probability of a transition between poor and non-poor health status (Figure 1).

Of the transitions with initial poor health status, 15.9% transitioned to recovery in the following year, and 84.1% remained in a poor health status. In contrast, of the transitions with a non-poor health status at the beginning, 32.4% ended with a poor health status the year after. 

As a consequence of all these transitions, among the 100 patients that reported poor health status at baseline, 21 (21.0%) recovered to a non-poor health status at least once during the follow-up period. Of the 37 patients that reported a non-poor health status at baseline, 15 (40.5%) reported a poor health status at least once during the follow-up period, and 20 (54.05%) reported a non-poor health status during the 2-year study period.

### 3.3. Predictors of Recovery from a Poor Health Status

We used three multivariate models to test potential predictors. Model 1, which included sociodemographic variables and BMI, suggested that participants with higher BMIs were less likely to achieve a non-poor health status the following year than those with lower BMIs. Model 2 included each of the non-pulmonary and pulmonary predictors and was adjusted for the variables in model 1. It showed that a non-poor health status was positively associated with being a current non-smoker, a higher percentage of FEV1, greater handgrip strength, and a 5STS ≥ 2; conversely, a non-poor health status was negatively associated with dyspnea ≥2. Finally, model 3 was adjusted for all the significant variables from models 1 and 2. It showed that current non-smoker behavior and greater handgrip strength were independent predictors of a transition to recovery from a poor health status for COPD patients (Table 2).

## 4. Discussion

This study determined that transitions to recovery from a poor health status occurred annually in nearly a fifth of patients with COPD. The most relevant independent predictors were current non-smoker status and handgrip strength. In addition, we found that nearly a third of transitions with non-poor health status at baseline finished with poor status. Moreover, over half the patients with non-poor health status at the beginning of the study remained in this state after 2 years. These results suggested that the nature of self-reported health status, measured with the CAT, was dynamic in patients with COPD.

To our knowledge, this longitudinal study was the first to find that both current non-smoker status and handgrip strength could predict recovery from a poor health status, measured with the CAT. Previous cross-sectional studies have identified these two determinants, but separately [7,20,21,22,23,24,25,26,27]. For example, Martínez et al. found that CAT scores greater than 10 points identified subjects that were current smokers or had smoked in the past [20]. Similarly, Brien et al. found a relationship between current smoker status and self-reported health status measured with the CAT [7]. This association could be explained by the inflammation produced by tobacco smoke, which causes an increase in several molecular inflammatory markers in subjects with COPD, including C-reactive protein, interleukin (IL)-6, IL-8, tumor necrosis factor–α, and fibrinogen, compared to healthy subjects [21,22]. As a result, lung function deteriorates, which leads to limitations in activities of daily living that require additional efforts, such as walking long distances, and consequently, a worse health status [23]. Moreover, some studies, including Tashkin et al., have shown that, when patients stopped smoking, the inflammation disappeared [24].

On the other hand, Ansari et al. found a relationship between health status in patients with COPD and handgrip strength, measured with the domain "impact" of St George’s Respiratory Questionnaire (SGRQ). They found a strong relationship between COPD exacerbation frequency and that domain, which is related to the activities of daily life that require handgrip strength [25]. Similarly, Inoue et al. found that the CAT score was inversely associated with handgrip strength [26]. It is well known that muscle dysfunction (muscular atrophy and weakness) is a recognized feature of COPD [27]; muscle dysfunction leads to inactivity, and consequently, a worse perception of health status and a negative prognosis [25]. 

Although the CAT is frequently used to identify health status in patients with COPD [23], to our knowledge, the present analysis was the first to describe annual transitions in perceived health status among adults with COPD. We observed that health status was partially dynamic because, although most patients maintained their initial health status, nearly a fifth of the subjects recovered from a poor health status. These recovery patterns were consistent with findings in some, but not all previous studies [1,28]. For example, Irie et al. studied the longitudinal evolution of the CAT in subjects with COPD and found that the CAT score was a stable measure, in most subjects, throughout the 3 years of follow-up [1]. On the other hand, Rassouli et al., observed in their cohort with COPD that 25% of the subjects displayed significant improvement, and 38% displayed stable evolution [28].

### 4.1. Implications for Practice and Research

Based on our findings that the health status of patients with COPD is dynamic, it is advisable to evaluate health status annually. Practices that classify subjects with a permanent health status can lead to false positives or negatives over time. The two predictors of health status found in the present study, smoking status and handgrip strength, are potentially modifiable with specific clinical strategies. Indeed, it is well known that handgrip strength can improve with muscular rehabilitation programs in patients with stable COPD [29,30]. Therefore, the early recognition of a deficit in handgrip strength could identify patients that might benefit from these programs by improving a poor health status. Moreover, our findings suggested that smoking cessation programs should be offered to smokers with COPD; encouraging patients to stop smoking completely could improve their health status [31].

In the present study, all predictive factors were only measured at baseline. In future, a more longitudinal analysis might evaluate whether associations between smoking status, handgrip strength, and CAT scores could also be observed in individual patients over time. That type of analysis might reveal whether there is a synchronous association between changes in the CAT score and changes in smoking status and handgrip strength; or alternatively, whether changes in the CAT score might be influenced by changes in smoking status or handgrip strength.

### 4.2. Strengths and Limitations of the Study

This study had several strengths. First, it was a longitudinal study that lasted two years, and transitions between poor and non-poor self-reported health status were measured with the CAT instrument at three points in time. The main advantage of a longitudinal analysis is that changes can be analyzed in individual patients. In addition, the prevalence of health status can be measured at more than one moment in time, which might have provided a more realistic estimate of prevalence. Second, our models included a wide variety of previously identified factors related specifically to the perception of health status in patients with COPD. In addition, the CAT was a standardized, previously validated questionnaire, which provided objective measures and allowed comparisons with other studies that used standardized, validated questionnaires.

Our study also had several limitations. First, although we included a wide variety of possible predictive factors of the CAT in our model, there could be other factors not included in our model that could have improved its predictive power (e.g., the quantity of drugs taken regularly, participation in rehabilitation programs, etc.) [32,33]. Second, although measurements were made at three time points (once per year) to determine the course of health status, additional transitions between these time points might have occurred. We chose three time point measurements specifically to optimize the capture of possible changes in health status measured with the CAT. Although little is known about the patterns of change between the different states of health, we assumed that most changes occurred annually [1,34]; therefore, we performed measurements at 1-year intervals, as previously described [1]. Third, data on rehabilitation services were lacking. Nevertheless, we know that rehabilitation services were not widely used by the patients. Finally, due to the small number of women in the cohort, the results in women must be interpreted with caution, because they might not be generalizable to all women with COPD. Nevertheless, in Spain, epidemiological studies show that the prevalence of COPD in women is lower and COPD is more underdiagnosed in women than in men. In 2007, the EPI-SCAN study determined that the prevalence of COPD in Spain was 15.1% in men and 5.7% in women [35]. Previously, in 1997, the epidemiological study of COPD in Spain (IBERPOC study) determined a prevalence of COPD of 14.3% in men and 3.9% in women [36].

## 5. Conclusions

This study showed that the perception of health status (measured with the CAT) was a dynamic factor in patients with COPD. We also showed that poor health status was common among patients with COPD, and that the transition towards recovery from poor health was more likely among patients that were current non-smokers and had greater handgrip strength, compared to other patients. Moreover, these findings supported the notion that the perception of health status could be determined with non-pulmonary factors, in addition to the respiratory state of the patient. 

## Figures and Tables

**Table 1:** Characteristics of the study population, grouped by health status at baseline (*n* = 137) ^a^.

Characteristics	All (*n* = 137)	Non-Poor Health Status (*n* = 37)	Poor Health Status (*n* = 100)	*p*-Value
Sociodemographic variables				
Age (years), mean ± SD	66.9 ± 8.3	65.9 ± 8.58	67.2 ± 8.24	0.394
Male	120 (87.6)	32 (86.5)	88 (88.0)	0.811
Non-pulmonary variables				
BMI (kg/m^2^), mean ± SD	28.92 ± 5.05	28.58 ± 5.40	29.04 ± 4.94	0.635
Number Comorbidities, mean ± SD	3.15 ± 1.64)	2.84 ± 1.62	3.26 ± 1.64	0.184
Heart disease (yes)	19 (13.9)	2 (5.4)	17 (17.0)	0.081
Depression (HAD-D ≥ 11)	13 (9.5)	0 (0)	13 (13.0)	0.021
Low physical activity	37 (27.0)	10 (27.0)	27 (27.0)	0.000
Handgrip strength, mean ±SD	28.30 ± 7.98	29.34 ± 8.13	27.91 ± 7.93	0.353
6-minute walk test (m), mean ± SD	349.13 ± 84.69	380.95 ± 68.53	336.99 ± 87.39	0.007
5STS ≥ 2	99 (72.3)	35 (94.6)	64 (64.0)	0.000
Pulmonary variables				
Smoking, mean pack-years ± SD	58.72 ± 25.46	51.08 ± 19.69	61.55 ± 26.83	0.015
Current non-smoker	96 (70.1)	28 (75.7)	68 (68.0)	0.384
CAT score, mean ± SD	14.19 (7.31)	6.03 (2.17)	17.21 (6.14)	0.000
Dyspnea (mMRC ≥ 2)	49 (35.8)	2 (5.4)	47 (47.0)	0.000
Exacerbations ^b^ ≥ 2	79 (57.7)	11 (29.7)	68 (68.0)	0.000
FEV1 (% predicted), mean ± SD	50.21 ± 16.49	58.16 ± 14.94	47.27 ± 16.13	0.000
GOLD Stage				0.000
A	24 (17.5)	23 (62.2)	1 (1.0)	
B	22 (16.1)	0 (0)	22 (22.0)	
C	12 (8.8)	12 (32.4)	0 (0)	
D	79 (57.7)	2 (5.4)	77 (77.0)	

SD, standard deviation; BMI, body mass index; HAD, Hospital Anxiety and Depression Scale; 5STS, 5 sit to-stand test; mMRC, modified British Medical Research Council; FEV1, forced expiratory volume in 1 s; GOLD, Global Initiative for Chronic Obstructive Pulmonary Disease. ^a^ Values represent the number (%) of participants in each group, unless otherwise noted. ^b^ Moderate or severe exacerbations in the previous year.

**Table 2:** Multivariate regression models show the predictive strengths of factors associated with a transition to recovery from a poor health status in the following year (*n* = 176 1-year transitions) ^a^.

Predictors	Model 1 ^b^	Model 2 ^c^	Model 3 ^d^
Sociodemographic variables			
Age (per year)	0.98 (0.94–1.03) 0.565		1.27 (0.96–1.08) 0.455
Female	1.67 (0.50–5.58) 0.40		4.17 (0.65–26.31) 0.129
Non-pulmonary variables			
BMI (kg/m^2^)	0.91 (0.85–0.99) 0.042		0.90 (0.81–1.00) 0.067
Number of comorbidities		1.03 (0.77–1.39) 0.808	
Heart disease (yes)		0.48 (0.10–2.27) 0.356	
Depression (HAD-D ≥ 11)		0.71 (0.15–3.26) 0.662	
Low physical activity		0.60 (0.21–1.70) 0.341	
Handgrip strength		1.07 (1.01–1.14) 0.035	1.08 (1.00–1.16) 0.049
6-minute walk test (m)		1.00 (0.99–1.00) 0.949	
5STS ≥ 2		2.31 (0.88–6.08) 0.089	1.88 (0.64–5.54) 0.247
Pulmonary variables			
Smoking, pack-years		0.99 (0.97–1.01) 0.826	
Current non-smoker		2.77 (0.92–8.33) 0.068	3.92 (1.11–13.73) 0.033
Dyspnea (mMRC ≥ 2)		0.33 (0.12–0.94) 0.038	0.40 (0.10–1.58) 0.195
Exacerbations ≥ 2		0.88 (0.35–2.25) 0.801	
FEV1 (% of predicted)		1.02 (0.99–1.05) 0.112	1.01 (0.98–1.04) 0.357

BMI, body mass index; HAD-D, Hospital Anxiety and Depression Scale; 5STS, 5 sit-to-stand test; mMRC, modified British Medical Research; FEV1, forced expiratory volume in 1 s. Values are odd ratios (95% confidence intervals) and P-value. ^a^ Includes all 1-year transitions in which the participant had a CAT score ≥ 10 the first year and continued to participate the following year ^b^ Model 1 includes sociodemographic variables. ^c^ Model 2 includes each predictor adjusted for age, sex, and BMI. ^d^ Model 3 is fully adjusted for significant variables from model 2 and age, sex, and BMI.
